# Are depression and suffering distinct? An empirical analysis

**DOI:** 10.3389/fpsyg.2022.970466

**Published:** 2022-09-02

**Authors:** Richard G. Cowden, Dorota Wȩziak-Białowolska, Eileen McNeely, Tyler J. VanderWeele

**Affiliations:** ^1^Human Flourishing Program, Institute for Quantitative Social Science, Harvard University, Cambridge, MA, United States; ^2^Sustainability and Health Initiative (SHINE), Department of Environmental Health, Harvard T. H. Chan School of Public Health, Boston, MA, United States; ^3^Centre for Evaluation and Analysis of Public Policies, Faculty of Philosophy, Jagiellonian University, Cracow, Poland

**Keywords:** depression, suffering, psychological distress, health, well-being

## Abstract

Depression and the subjective experience of suffering are distinct forms of distress, but they are sometimes commingled with one another. Using a cross-sectional sample of flight attendants (*n* = 4,652), we tested for further empirical evidence distinguishing depression and suffering. Correlations with 15 indices covering several dimensions of well-being (i.e., physical health, emotional well-being, psychological well-being, character strengths, social well-being, financial/material well-being) indicated that associations with worse well-being were mostly stronger for depression than suffering. There was a large positive correlation between depression and suffering, but we also found evidence of notable non-concurrent depression and suffering in the sample. After dividing participants into four groups that varied based on severity of depression and suffering, regression analyses showed higher levels of well-being among those with both none-mild depression and none-mild suffering compared to those with moderate-severe depression, moderate-severe suffering, or both. All indices of well-being were lowest among the group of participants with moderate-severe depression and moderate-severe suffering. In addition to providing further evidence supporting a distinction between depression and suffering, our findings suggest that concurrent depression and suffering may be more disruptive to well-being than when either is present alone.

## Introduction

Depression is the 13th leading cause of disability (Vos et al., 2020) and the most common mental health condition globally (World Health Organization, 2017). Numerous theories, treatments, and initiatives have been advanced to understand the etiology of depression, support those afflicted with the condition, and prevent its onset among high-risk populations (Callahan and Berrios, 2005). Although substantial progress has been made in these (and other) areas of the scientific literature on depression, a complex component to classifying depression is setting thresholds that clinicians must apply when evaluating the complex, multidimensional signs and symptoms of depression that might present in healthcare settings (Clark et al., 2017). Accurate classification of depression necessarily involves ruling out alternative mental health conditions, substances, medical illnesses, and other conditions that do not currently receive formal diagnoses.

One form of distress that has proven challenging to distinguish from depression is suffering, which might be understood as an undesired experiential state that involves bearing under the loss or privation of some perceived good (VanderWeele, 2019). For example, some proposed definitions of suffering specify one or more signs of depression (e.g., hopelessness) under the banner of suffering (e.g., Best et al., 2015), and there are instances in which assessment of suffering has been conflated with symptoms of depression (e.g., Schulz et al., 2009). Suffering can be challenging to differentiate from depression because both are laden with negative affect, but a person is only thought to be suffering when “she has an unpleasant or negative affective experience that she minds, where to mind some state is to have an occurrent desire that it not be occurring” (Brady, 2018, p. 27). Similar to research that has documented an absence of self-reported suffering among some individuals who endorse severe pain (e.g., Body et al., 2015), this view suggests that a person may meet diagnostic criteria for depression but not endorse suffering. More accurate discernment between depression and suffering at the levels of conceptualization and assessment could be advantageous to practitioners who must decide whether a client’s depression symptoms are sufficiently impairing to warrant classification as major depression, which is an important decision because of the many ramifications—both positive (e.g., access to supportive services) and negative (e.g., social stigma)—that can accompany formal diagnosis of a mental disorder (Clark et al., 2017). It would also contribute to developing a clearer understanding of the implications of suffering for individual well-being, both in the absence of depression and when depression symptoms are more severe. In the present study, we test for further evidence empirically distinguishing depression and suffering.

### Conceptual distinctions between depression and suffering

Although depression and suffering share qualities that can make it difficult to practically distinguish a major depressive episode from an experiential state of suffering (e.g., both are negatively valanced conditions that can be disruptive to various domains of life), depression and suffering can be differentiated on conceptual grounds. We provide a non-exhaustive overview of some key areas in which depression and suffering might be distinguished conceptually.

Depression is a mood disorder that is determined by evaluating symptoms against a standard set of widely used diagnostic criteria. There are many qualitatively different ways in which people may qualify for the same diagnostic criterion (Fried et al., 2020), but clear parameters are provided in diagnostic classification systems (e.g., Diagnostic and Statistical Manual of Mental Disorders, 5th Edition) about the type and number of symptoms that are required for diagnosis of depression. In contrast to the objective criteria that are applied to diagnose depression, suffering is a *subjective* experiential state that can only be established by the individual who is experiencing it (Tate and Pearlman, 2019; VanderWeele, 2019). Hence, suffering is not restricted (and perhaps not reducible) to a prespecified set of diagnostic criteria (Cowden et al., 2022), and between-person experiences of suffering can vary considerably based on the unique, person-specific lens that shapes the way in which individuals interpret, label, and respond to internal and external stimuli (Cassell, 1999; Cowden et al., 2021a).

Clinical depression principally involves low mood and/or anhedonia, coupled with a combination of at least four secondary symptoms (e.g., feelings of worthlessness, suicidal ideation, and sleep disturbance) that intersect emotional, cognitive, behavioral, and physical dimensions of functioning (Clark et al., 2017). Although suffering also has a permeating quality that impacts multiple domains of a person’s life, depressed mood and/or markedly diminished interest or pleasure in usual activities may not be core (or even ancillary) features of a person’s experience of suffering. For example, many theoretical accounts suggest that a central characteristic of suffering is the threat to, or loss of, one’s sense of personhood (e.g., Cassell, 1999; Tate and Pearlman, 2019). The physical dimension of depression (i.e., somatic symptoms, such as fatigue) resonates particularly closely with accounts of suffering that are prevalent in the empirical literature, given that research on suffering has typically addressed clinical populations dealing with physical health concerns or illness (Cowden et al., 2021b). However, even if physical symptoms or issues are the primary cause of a person’s suffering, the object of their suffering may be entirely different (VanderWeele, 2019).

Both depression and suffering share qualities of intensity and persistence. For example, depression is diagnosed if symptoms have been present for at least two consecutive weeks and they cause clinically significant distress or impairment in psychosocial functioning (American Psychiatric Association, 2013), whereas suffering involves enduring a negative experience that is considerably intense or persistent (VanderWeele, 2019). However, depression and suffering can often be differentiated based on the object of suffering, as the object of a person’s suffering may be quite distinct from their symptoms of depression (Cowden et al., 2021b). In the case of bereavement, losing a loved one may be the cause of both depression and suffering, but the object of the person’s suffering may be their sense of loneliness rather than their depression symptoms. Such a distinction is important because it could inform decisions about treatment approaches that will effectively address depression and suffering when both are present.

### Empirical evidence distinguishing depression and suffering

If depression and suffering are not distinct phenomena, a reasonable expectation is that research would find associations between measures of depression and suffering that demonstrate a high degree of convergence. However, the findings of previous studies provide little evidence in alignment with this notion. Effect sizes reported in a number of prior cross-sectional studies suggest a small-to-moderate positive association between depression and suffering (e.g., Büchi et al., 2002; Brady et al., 2019; Davis et al., 2021), with slightly smaller effect sizes found in a few longitudinal studies that have been conducted (e.g., Cowden et al., 2021b; Ho et al., 2022).

Some findings indicate that individuals may experience a severe degree of suffering in the absence of clinical depression, and there are people with depression who report mild or even no suffering. In one cross-sectional study of 381 advanced cancer patients, Wilson et al. (2007) found that 56.1% of the participants who reported moderate-extreme suffering did not have depression. Of those who reported none-mild suffering, 12.7% qualified for diagnosis of depression. Therefore, suffering and depression may not always occur contemporaneously.

Providing further evidence of a distinction between depression and suffering, measures of each construct often diverge in the strength of their associations with other variables. In previous studies that have reported associations of both depression and suffering with auxiliary variables, effect sizes corresponding with depression have been quite different compared to those found for suffering. For example, the findings of several studies indicate that depression is generally more strongly correlated with anxiety than suffering, whereas correlations of suffering with other criterion variables (e.g., perceived posttraumatic growth, pain intensity) have been stronger than those reported for depression (e.g., Büchi et al., 2009; Streffer et al., 2009).

Other emerging evidence suggests that suffering may be disruptive to well-being above and beyond depression. To illustrate, a recent longitudinal study of 184 adults with chronic illness found that suffering was associated with worse subsequent psychological well-being after adjusting for a range of covariates, including depression (Cowden et al., 2021b). Such findings underscore the potential for suffering to uniquely impact well-being in ways that may not be accounted for by depression. However, further research is needed to ascertain whether suffering that co-exists with depression is associated with worse well-being compared to when either is present alone, which dimensions of well-being tend to be impacted more severely by co-occurring depression and suffering, and the mechanisms involved. Evidence along these lines could prove useful to the development and implementation of interventions to support people who have depression and are concurrently suffering.

### The present study

To date, much of the research on suffering has focused on clinical samples comprising older adults who are experiencing physical health symptoms (e.g., pain), have chronic medical illnesses (e.g., cancer), or are receiving palliative care (Cowden et al., 2021b). However, recent research has documented the potential for suffering to degrade well-being among non-clinical young adult populations (see Ho et al., 2022), suggesting that further consideration should be given to the implications of suffering for the functioning of both clinical and non-clinical populations. Broadening the scope of research on suffering to a more diverse range of populations could enhance our understanding about the nature of suffering, its linkages to other related constructs (e.g., depression), and how it might be effectively addressed to promote well-being. To these ends, this study extends the current body of empirical literature on suffering to a non-clinical sample of currently employed flight attendants, whose employment involves exposure to a variety of adverse working conditions (e.g., circadian rhythm disruption, high levels of occupational noise) and a demanding job role (e.g., managing one’s emotion in alignment with organizational goals) that can heighten risk of distress and other health-related consequences (Węziak-Białowolska et al., 2020). Thus, flight attendants are an important population in which to study suffering and its implications for well-being.

There were three core purposes guiding this study. First, we explored whether depression symptoms and subjective experiences of suffering differ in the strength of their associations with indices of well-being by estimating bivariate associations of each with 15 criterion variables that tap into several dimensions of well-being (i.e., physical health, emotional well-being, psychological well-being, character strengths, social well-being, financial/material well-being). We anticipated that both depression and suffering would be associated with worse well-being, but that there would be some variation in effect sizes that emerged for each. Second, we tested for further evidence of non-concurrent depression and suffering by estimating co-occurring categorical combinations of depression symptoms (i.e., none-minimal, mild, moderate, severe) and suffering (i.e., none, mild, moderate, severe). Consistent with conceptual and empirical literature, we expected to find some evidence of mild, moderate, and severe depression in the absence of suffering, as well as mild, moderate, and severe suffering when depression symptoms were none-minimal. Third, we generated four groups that varied based on severity of both depression and suffering to examine whether combinations of moderate-severe depression and none-mild suffering, moderate-severe suffering and none-mild depression, and moderate-severe depression and moderate-severe suffering are associated with worse well-being compared to none-mild depression and none-mild suffering. Based on the understanding that depression and suffering are distinct forms of distress, we anticipated that well-being would be lowest among participants with moderate-severe depression and moderate-severe suffering. In a supplementary analysis that focused on the subsample of participants who reported moderate-severe depression, we examined the associations of four groups that varied based on severity of suffering (i.e., none, mild, moderate, severe) with the indices of well-being. We expected that well-being would be lowest among participants with moderate-severe depression and severe suffering.

## Materials and methods

### Study sample

This study used the third wave of data from the Flight Attendant Health Study (FAHS), which is an ongoing health surveillance study of flight attendants who are employed mostly by airlines operating within the United States, United Kingdom, and Canada. Further details about the purpose, scope, and design of the FAHS can be found elsewhere (see McNeely et al., 2014; Węziak-Białowolska et al., 2020). We selected the third wave of data for this study because it was the only wave that included a measure of subjective suffering.

The third wave of data collection occurred between 2017 and 2018. Current and former flight attendants who were previously enrolled in the FAHS were invited participate in the third wave of data collection. Additional flight attendants were recruited via national and local unions that represented workers in the airline industry, postcards that were sent directly to flight attendants who were employed by selected airlines, and in-person recruitment campaigns that were conducted on airport premises. Participants were entered into a lottery draw to win one of 10 Amazon gift cards (each valued at $99).

All prospective participants were directed to an online survey via a secure weblink. Those who were interested in participating first provided electronic consent and then responded to the self-administered survey. The survey items covered a wide range of topics related to health, work, and quality of life. A total of *N* = 10,378 participants completed the Wave 3 survey before the end of 2018. Of those, *n* = 7,270 participants were currently employed flight attendants who had been a crew member of at least one flight within the last 12 months. Consistent with prior studies that have used the FAHS dataset (e.g., Węziak-Białowolska et al., 2020), we restricted our analyses to the subsample (*n* = 4,652) of currently employed flight attendants who had complete cases on the primary study variables (including covariates).

The sociodemographic characteristics of the full analytic sample are reported in Table 1. At least half of the participants resided in the United States (52.09%), with a smaller number of participants from the United Kingdom (6.58%), Canada (4.94%), and other countries around the world (2.75%; missing = 33.64%). Participants (*M*_age_ = 48.08, *SD* = 12.12) were mostly female (78.93%), heterosexual (83.51%), and self-identified as White (81.19%). All participants had completed some postsecondary education or higher. Slightly more than half of the sample was not married (54.32%), and most participants did not have child (78.16%) or adult/elderly dependents (87.70%).

**TABLE 1:** Sociodemographic characteristics of participants in the full analytic sample (*n* = 4,652).

Characteristic	*n* (%)	*M* ± *SD*
Age (years)		48.08 ± 12.12
**Country of residence**		
United States	2,423 (52.09)	
United Kingdom	306 (6.58)	
Canada	230 (4.94)	
Other	128 (2.75)	
Missing	1,565 (33.64)	
**Gender**		
Female	3,672 (78.93)	
Male	977 (21.00)	
Other	3 (0.06)	
**Sexual orientation**		
Heterosexual	3,885 (83.51)	
Other	767 (16.49)	
**Racial/ethnic status**		
White	3,777 (81.19)	
Non-white	875 (18.81)	
**Marital status**		
Married	2,125 (45.68)	
Other	2,527 (54.32)	
**Educational attainment**		
Some postsecondary education or higher	4,652 (100.00)	
**Child dependents**		
No	3,636 (78.16)	
Yes	1,016 (21.84)	
**Adult/elderly dependents**		
No	4,080 (87.70)	
Yes	572 (12.30)	

M, mean; SD, standard deviation. Cumulative percentages may not add up to 100% due to rounding.

### Measures

#### Depression

Participants completed the Patient Health Questionnaire-9 (PHQ-9; Kroenke et al., 2001). The PHQ-9 comprises nine items that assess the severity of depression symptoms experienced during the last two weeks. The items are rated using a four-point response format (0 = Not at all; 3 = Nearly every day). Responses to each item are summed for a total score ranging from 0 to 27. In this study, the estimated internal consistency of scores on the PHQ-9 was α = 0.87.

#### Suffering

We used a single item from the Personal Suffering Assessment (VanderWeele, 2019) to assess global suffering (i.e., “To what extent are you suffering?”). The item is rated using an 11-point response format (0 = Not suffering at all; 10 = Suffering terribly).

#### Criterion variables

We used a range of single-item criterion variables taken from two well-validated and widely used measures: the Centers for Disease Control and Prevention’s 14-item health-related quality of life measure (Centers for Disease Control and Prevention, 2000; see also Moriarty et al., 2003) and the Secure Flourishing Index (VanderWeele, 2017; see also Höltge et al., 2022). Criterion variables were limited to items that were available in Wave 3 of the FAHS and selected to canvas well-being as broadly as possible. Items tapped into dimensions of physical health (i.e., general health, physically unhealthy days, disability days, vitality days, sleepless days), emotional well-being (i.e., mentally unhealthy days, happiness, life satisfaction), psychological well-being (i.e., meaning in life, sense of purpose), character strengths (i.e., promote good, delay gratification), social well-being (i.e., satisfying relationships), and financial/material well-being (i.e., financial stability, material stability). The items and corresponding response options for each can be found in Supplementary Table 1.

### Statistical processing

An initial set of descriptive analyses were performed to explore the association between depression symptom severity and suffering. Using continuous scores of each variable, we began by computing the zero-order (Pearson) correlation between them. For comparative purposes, we also estimated the zero-order correlations of both depression symptom severity and suffering with each of the criterion variables.

To ascertain the shared distribution of depression symptom severity and suffering, we recoded both variables into categorical variables comprising four groups each. Drawing on prior studies (e.g., Rein et al., 2021; Rothermich et al., 2021), we grouped depression symptoms into *depression severity* categories of none-minimal (0−4), mild (5−9), moderate (10−14), and severe symptoms (15−27). For consistency purposes and to simplify interpretation, we divided suffering into *suffering severity* groups that roughly corresponded with those used to categorize severity of depression symptoms: none (0), mild (1−3), moderate (4−6), and severe suffering (7−10). We used these categorical variables to determine the prevalence of (a) suffering at different levels of depression symptom severity and (b) depression symptom severity at different levels of suffering.

Our primary analysis involved estimating associations between combinations of depression and suffering severity and the well-being criterion variables. Before proceeding, we sought to conserve statistical power and facilitate interpretation of the results by condensing the four categories of both the depression severity and suffering severity groups into two groups each. We merged the two categories with the lowest depression severity (i.e., none-minimal and mild) into a none-mild group (corresponding with PHQ-9 scores ranging from 0 to 9), and the two categories with the highest depression severity (i.e., moderate and severe) were combined into a moderate-severe group (corresponding with PHQ-9 scores ranging from 10 to 27). This categorization approach aligns with the cutoff score (i.e., ≥10) that is widely used to screen for possible depression (Levis et al., 2019). Similarly, the two categories with the lowest severity of suffering (i.e., none and mild) were merged into a none-mild group (corresponding with suffering scores ranging from 0 to 3), and the two categories with the highest severity of suffering (i.e., moderate and severe) were combined into a moderate-severe group (corresponding with suffering scores ranging from 4 to 10). These modified depression and suffering severity variables were combined to create a *depression-suffering severity* variable comprising four groups: (a) none-mild depression and none-mild suffering (*n* = 2,477), (b) moderate-severe depression and none-mild suffering (*n* = 450), (c) moderate-severe suffering and none-mild depression (*n* = 910), and (d) moderate-severe depression and moderate-severe suffering (*n* = 815). We then used the depression-suffering severity variable in a series of linear regression analyses to determine whether the groups with moderate-severe depression, moderate-severe suffering, or both tended to score lower on the indices of well-being compared to the none-mild depression and none-mild suffering group (reference group). Models adjusted for age (continuous), gender (female/other vs. male), sexual orientation (heterosexual vs. other), racial/ethnic status (White vs. other), marital status (married vs. other), child dependents (no vs. yes), and adult/elderly dependents (no vs. yes). Given variations in recommendations and practices of correcting for multiple testing, we report the statistical significance (*p* < 0.05) of associations relative to the reference group both before and after applying Bonferroni corrections based on the number of analyses performed with the criterion variables. The *p*-value cutoff after Bonferroni correction was 0.0033 (α = 0.05/15). Estimated marginal means are reported for all four groups, with Cohen’s *d* used to present standardized differences between the reference group (i.e., none-mild depression and none-mild suffering) and each of the other groups.

We supplemented the primary analysis with a series of regression models in which each of the well-being indices was regressed on depression severity (none-mild depression = 0 vs. moderate-severe depression = 1), suffering severity (none-mild suffering = 0 vs. moderate-severe suffering = 1), and the interaction between them (one criterion variable at a time). Models included the same set of covariates indicated above. Using the procedure outlined in VanderWeele and Tchetgen (2014), we decomposed the joint associations of depression severity and suffering severity into the proportions of each association that were attributable to three components: that attributable to depression severity, that attributable to suffering severity, and that due to their interaction. This enabled us to evaluate whether the interactions of depression severity and suffering severity are roughly additive (i.e., no evidence of an interaction), subadditive (i.e., the joint association of depression and suffering is smaller than the sum of their independent effects), or superadditive (i.e., the joint association of depression and suffering is larger than the sum of their independent associations), which can be practically useful in determining which predictor variable may be the most appropriate to target for intervention (VanderWeele and Knol, 2014).

The primary analysis was complemented by a secondary analysis involving the subsample of participants who reported moderate-severe depression symptoms (*n* = 1,265). We created a *depression and graded suffering* variable comprising four groups of participants that were distinguishable by suffering severity: (a) moderate-severe depression and no suffering (*n* = 76), (b) moderate-severe depression and mild suffering (*n* = 374), (c) moderate-severe depression and moderate suffering (*n* = 500), and (d) moderate-severe depression and severe suffering (*n* = 315). We used this depression and graded suffering variable to perform a series of linear regression analyses estimating whether the groups with moderate-severe depression symptoms in combination with mild, moderate, or severe suffering tended to report worse well-being on the criterion variables compared to the group with moderate-severe depression symptoms that occur in the absence of suffering (reference group). Models adjusted for the same set of covariates that were included in the primary analysis, and we corrected for multiple testing using the same approach described above. Emulating the primary analysis, we report the estimated marginal means for all four groups and use Cohen’s *d* to present standardized differences between the reference group (moderate-severe depression and no suffering) and each of the other groups.

## Results

Zero-order correlations involving severity of depression symptoms and suffering are reported in Table 2. There was a large positive association between depression and suffering, *r* = 0.50, *p* < 0.001. Both depression and suffering were associated with worse well-being on all criterion variables. Correlations were generally larger for depression (*r*s = |0.26 to 0.62|) relative to those found for suffering (*r*s = |0.09 to 0.56|). Depression was most strongly associated with indices of emotional well-being (i.e., mentally unhealthy days, happiness, life satisfaction), whereas the strongest correlations for suffering were found with selected indices of physical health (i.e., general health, physically unhealthy days, disability days). Depression and suffering both evidenced their weakest associations with the character strengths indices.

**TABLE 2:** Zero-order correlations of depression symptoms and suffering with criterion variables (*n* = 4,652).

Variable	*M* ± *SD*	Depression symptoms	Suffering
Depression symptoms	6.78 ± 5.25		
Suffering	2.90 ± 2.61	0.50 [0.48, 0.52]***	
**Physical health**			
General health	3.59 ± 0.90	−0.44 [−0.47, −0.42]***	−0.49 [−0.51, −0.47]***
Physically unhealthy days	5.02 ± 7.65	0.26 [0.23, 0.28]***	0.51 [0.49, 0.54]***
Disability days	4.11 ± 6.99	0.43 [0.41, 0.45]***	0.56 [0.54, 0.58]***
Vitality days	11.68 ± 9.12	−0.53 [−0.55, −0.51]***	−0.40 [−0.42, −0.37]***
Sleepless days	11.69 ± 8.53	0.39 [0.37, 0.42]***	0.25 [0.22, 0.28]***
**Emotional well-being**			
Mentally unhealthy days	5.68 ± 7.68	0.61 [0.59, 0.63]***	0.48 [0.46, 0.51]***
Happiness	7.08 ± 1.84	−0.62 [−0.64, −0.60]***	−0.39 [−0.41, −0.36]***
Life satisfaction	7.07 ± 1.98	−0.59 [−0.61, −0.57]***	−0.43 [−0.46, −0.41]***
**Psychological well-being**			
Meaning in life	7.48 ± 2.02	−0.52 [−0.54, −0.50]***	−0.30 [−0.32, −0.27]***
Sense of purpose	6.85 ± 2.44	−0.47 [−0.49, −0.44]***	−0.22 [−0.25, −0.19]***
**Character strengths**			
Promote good	7.91 ± 1.62	−0.24 [−0.27, −0.21]***	−0.09 [−0.12, −0.06]***
Delay gratification	7.22 ± 1.89	−0.21 [−0.24, −0.18]***	−0.10 [−0.13, −0.07]***
**Social well**-**being**			
Satisfying relationships	6.29 ± 2.61	−0.47 [−0.49, −0.44]***	−0.25 [−0.28, −0.23]***
**Financial and material well-being**			
Financial stability	5.65 ± 3.23	−0.38 [−0.40, −0.35]***	−0.26 [−0.29, −0.23]***
Material stability	6.51 ± 3.07	−0.39 [−0.41, −0.36]***	−0.28 [−0.30, −0.25]***

M, mean; SD, standard deviation. 95% confidence intervals for Pearson correlations in brackets. ***p < 0.001.

Cross-tabulations of *depression severity* and *suffering severity* are reported in Table 3. Within the categories of suffering severity, there was a trend toward higher percentages of participants endorsing mild, moderate, or severe depression symptoms as the severity of reported suffering increased. A similar pattern was found within categories of depression severity, such that higher percentages of participants tended to report mild, moderate, or severe suffering as the severity of depression symptoms increased. The general findings from this set of analyses suggest that depression and suffering are strongly associated with one another, but there was also evidence demonstrating that experiences of depression and suffering are distinct and can occur independently. Specifically, almost 30% of participants who indicated they were not suffering at all endorsed mild (20.64%), moderate (5.14%), or severe depression symptoms (1.26%). In addition, considerable percentages of participants in the mild (39.25%), moderate (23.59%), and severe suffering categories (14.70%) had none-minimal symptoms of depression. Within categories of depression severity, more than 50% of those in the none-minimal depression symptoms group were categorized as suffering mildly (35.82%), moderately (14.53%), or severely (4.25%). We also found non-trivial percentages of participants in the mild (16.55%), moderate (7.24%), and severe depression categories (3.55%) who reported that they were not suffering at all.

**TABLE 3:** Summary statistics for depression and suffering severity categories (*n* = 4,652).

	Suffering severity			
	None (*n* = 1,187)	Mild (*n* = 1,740)	Moderate (*n* = 1,174)	Severe (*n* = 551)
Depression symptoms, *M* ± *SD* (range)	3.36 ± 3.32 (0−23)	6.23 ± 4.16 (0−22)	9.02 ± 5.42 (0−26)	11.10 ± 6.04 (0−27)
**Depression severity**				
None-minimal (0−4)	866 (72.96%)	683 (39.25%)	277 (23.59%)	81 (14.70%)
Mild (5−9)	245 (20.64%)	683 (39.25%)	397 (33.82%)	155 (28.13%)
Moderate (10−14)	61 (5.14%)	306 (17.59%)	308 (26.24%)	167 (30.31%)
Severe (15−27)	15 (1.26%)	68 (3.91%)	192 (16.35%)	148 (26.86%)

	**Depression severity**			
	**None-minimal (*n* = 1,907)**	**Mild (*n* = 1,480)**	**Moderate (*n* = 842)**	**Severe (*n* = 423)**

Suffering, *M* ± *SD* (range)	1.67 ± 2.14 (0−10)	3.03 ± 2.38 (0−10)	4.13 ± 2.43 (0−10)	5.53 ± 2.39 (0−10)
**Suffering severity**				
None (0)	866 (45.41%)	245 (16.55%)	61 (7.24%)	15 (3.55%)
Mild (1−3)	683 (35.82%)	683 (46.15%)	306 (36.34%)	68 (16.08%)
Moderate (4−6)	277 (14.53%)	397 (26.82%)	308 (36.58%)	192 (45.39%)
Severe (7−10)	81 (4.25%)	155 (10.47%)	167 (19.83%)	148 (34.99%)

M, mean; SD, standard deviation. Cumulative percentages may not add up to 100% due to rounding.

Summary statistics for the regression models involving categories of *depression-suffering severity* are reported in Table 4. The results supported robust differences between the none-mild depression and none-mild suffering group (reference group) and two or more of the comparison groups on all indices of well-being, with the pattern of results consistently supporting higher levels of well-being among the none-mild depression and none-mild suffering group. The moderate-severe depression and moderate-severe suffering group evidenced the largest differences from the none-mild depression and none-mild suffering group on all criterion variables. The strongest associations were found for several indices of physical health (i.e., general health, physically unhealthy days, disability days, vitality days), emotional well-being (i.e., mentally unhealthy days, happiness, life satisfaction), and psychological well-being (i.e., meaning in life).

**TABLE 4:** Estimated marginal means, standard errors, and between-subjects effect sizes for contrasts with the none-mild depression and none-mild suffering group (*n* = 4,652).

Criterion	None-mild depression and none-mild suffering (*n* = 2,477)	Moderate-severe depression and none-mild suffering (*n* = 450)	Moderate-severe suffering and none-mild depression (*n* = 910)	Moderate-severe depression and moderate-severe suffering (*n* = 815)
**Physical health**				
General health				
*EMM* ± *SE*	3.93 ± 0.03	3.51 ± 0.04***	3.29 ± 0.03***	2.85 ± 0.03***
Cohen’s *d* [95% CI]^a^	−	−0.30 [−0.36, −0.24]	−0.60 [−0.66, −0.54]	−0.97 [−1.03, −0.91]
Physically unhealthy days				
*EMM* ± *SE*	2.04 ± 0.23	3.28 ± 0.37***	8.60 ± 0.30***	9.74 ± 0.30***
Cohen’s *d* [95% CI]^a^	−	0.10 [0.04, 0.16]	0.72 [0.66, 0.78]	0.81 [0.75, 0.87]
Disability days				
*EMM* ± *SE*	0.74 ± 0.20	2.78 ± 0.33***	5.75 ± 0.26***	10.28 ± 0.26***
Cohen’s *d* [95% CI]^a^	−	0.19 [0.14, 0.25]	0.63 [0.57, 0.69]	1.15 [1.09, 1.30]
Vitality days				
*EMM* ± *SE*	15.18 ± 0.27	7.70 ± 0.44***	10.06 ± 0.35***	5.07 ± 0.35***
Cohen’s *d* [95% CI]^a^	−	−0.52 [−0.58, −0.47]	−0.48 [−0.54, −0.42]	−0.90 [−0.96, −0.84]
Sleepless days				
*EMM* ± *SE*	9.90 ± 0.27	15.07 ± 0.44***	12.40 ± 0.35***	16.87 ± 0.35***
Cohen’s *d* [95% CI]^a^	−	0.36 [0.31, 0.42]	0.23 [0.18, 0.29]	0.62 [0.57, 0.68]
**Emotional well-being**				
Mentally unhealthy days				
*EMM* ± *SE*	2.28 ± 0.21	7.03 ± 0.34***	5.32 ± 0.27***	14.26 ± 0.27***
Cohen’s *d* [95% CI]^a^	−	0.43 [0.37, 0.49]	0.37 [0.31, 0.42]	1.38 [1.31, 1.44]
Happiness				
*EMM* ± *SE*	7.77 ± 0.05	6.17 ± 0.08***	7.15 ± 0.07***	5.29 ± 0.07***
Cohen’s *d* [95% CI]^a^	−	−0.58 [−0.64, −0.53]	−0.30 [−0.36, −0.25]	−1.15 [−1.21, −1.09]
Life satisfaction				
*EMM* ± *SE*	7.95 ± 0.06	6.28 ± 0.09***	7.06 ± 0.07***	5.34 ± 0.07***
Cohen’s *d* [95% CI]^a^	−	−0.56 [−0.62, −0.50]	−0.40 [−0.46, −0.34]	−1.11 [−1.18, −1.05]
**Psychological well-being**				
Meaning in life				
*EMM* ± *SE*	8.35 ± 0.06	6.89 ± 0.10***	7.89 ± 0.08***	6.18 ± 0.08***
Cohen’s *d* [95% CI]^a^	−	−0.46 [−0.52, −0.40]	−0.19 [−0.25, −0.14]	−0.87 [−0.93, −0.81]
Sense of purpose				
*EMM* ± *SE*	7.85 ± 0.07	6.06 ± 0.12***	7.46 ± 0.10***	5.76 ± 0.10***
Cohen’s *d* [95% CI]^a^	−	−0.46 [−0.52, −0.41]	−0.13 [−0.19, −0.08]	−0.69 [−0.75, −0.63]
**Character strengths**				
Promote good				
*EMM* ± *SE*	8.28 ± 0.05	7.68 ± 0.09***	8.18 ± 0.07	7.63 ± 0.07***
Cohen’s *d* [95% CI]^a^	−	−0.22 [−0.27, −0.16]	−0.05 [−0.11, 0.01]	−0.30 [−0.35, −0.24]
Delay gratification				
*EMM* ± *SE*	7.61 ± 0.06	6.94 ± 0.10***	7.33 ± 0.08***	6.92 ± 0.08***
Cohen’s *d* [95% CI]^a^	−	−0.20 [−0.26, −0.15]	−0.11 [−0.17, −0.06]	−0.27 [−0.32, −0.21]
**Social well-being**				
Satisfying relationships				
*EMM* ± *SE*	6.99 ± 0.08	5.10 ± 0.13***	6.44 ± 0.10***	4.66 ± 0.10***
Cohen’s *d* [95% CI]^a^	−	−0.45 [−0.51, −0.39]	−0.18 [−0.23, −0.12]	−0.71 [−0.77, −0.65]
**Financial and material well-being**				
Financial stability				
*EMM* ± *SE*	6.23 ± 0.10	4.69 ± 0.16***	5.23 ± 0.13***	3.84 ± 0.13***
Cohen’s *d* [95% CI]^a^	−	−0.30 [−0.35, −0.24]	−0.25 [−0.31, −0.20]	−0.59 [−0.64, −0.53]
Material stability				
*EMM* ± *SE*	7.12 ± 0.10	5.61 ± 0.16***	6.10 ± 0.12***	4.66 ± 0.12***
Cohen’s *d* [95% CI]^a^	−	−0.30 [−0.36, −0.24]	−0.27 [−0.33, −0.21]	−0.63 [−0.68, −0.57]

CI, confidence interval; EMM, estimated marginal mean; SE, standard error. Models adjusted for age, gender, sexual orientation, racial/ethnic status, marital status, child dependents, and adult/elderly dependents.

^a^The none-mild depression and none-mild suffering group served as the reference group in each model.

***Comparison group differs significantly from the none-mild depression and none-mild suffering group at p < 0.05 both before and after Bonferroni correction (the p-value cutoff for Bonferroni correction was 0.05/15 = 0.0033 for each criterion variable).

Slightly weaker associations were found for the differences between the moderate-severe depression and none-mild suffering group and the none-mild depression and none-mild suffering group on the criterion variables (see Table 4). Associations were strongest for selected indices of emotional well-being (i.e., happiness, life satisfaction) and physical health (i.e., vitality days). Except for three indices of physical health (i.e., general health, physically unhealthy days, disability days), differences between the moderate-severe suffering and none-mild depression group and the none-mild depression and none-mild suffering group on the criterion variables were the smallest of any comparison group. These two groups evidenced the largest differences on the abovementioned indices of physical health, and the differences on these criterion variables were larger than those that emerged when the moderate-severe depression and none-mild suffering group was compared with the none-mild depression and none-mild suffering group.

Results for the decomposition of the joint associations of depression severity and suffering severity into their independent contributions and an interactive component for all indices of well-being are reported in Supplementary Table 2. The proportions of the associations that were attributable to depression severity alone, suffering severity alone, and their interaction varied across the criterion variables. In all cases, the proportion of the association attributable to depression severity and suffering severity on their own was larger than the proportion attributable to the interaction between them. Except for three indices of physical health (i.e., general health, physically unhealthy days, disability days), the proportions of the associations attributable to depression severity was larger than those found for suffering severity.

We present the summary statistics for the regression models involving categories of *depression and graded suffering* in Supplementary Table 3. The general pattern of results indicated that well-being was higher among the moderate-severe depression and no suffering group (reference group) compared to each of the other groups in which moderate-severe depression co-existed with mild, moderate, or severe suffering. Both the moderate-severe depression and moderate suffering group and the moderate-severe depression and severe suffering group demonstrated robust differences from the moderate-severe depression and no suffering group on indices of physical health (i.e., general health, physically unhealthy days, disability days, vitality days), emotional well-being (i.e., mentally unhealthy days, happiness), psychological well-being (i.e., meaning in life), and financial/material well-being (i.e., material stability). Except for meaning in life, differences on these criterion variables were larger for comparisons involving the moderate-severe depression and severe suffering group. Robust differences were also found between the moderate-severe depression and severe suffering group and the moderate-severe depression and no suffering group on life satisfaction and financial stability.

Both the moderate-severe depression and moderate suffering group and the moderate-severe depression and severe suffering group evidenced a modest difference from the moderate-severe depression and no suffering group on sleepless days. Modest differences between the moderate-severe depression and moderate suffering group and the moderate-severe depression and no suffering group were also found on life satisfaction, sense of purpose, and financial stability. The moderate-severe depression and mild suffering group also differed modestly from the moderate-severe depression and no suffering group on selected indices of physical health (i.e., general health, physically unhealthy days, disability days, vitality days) and psychological well-being (i.e., meaning in life). However, none of the comparisons that yielded more modest differences remained statistically significant after Bonferroni correction.

## Discussion

Conceptual literature and some previous empirical evidence support a distinction between depression and suffering, but they are sometimes conflated with one another in language use and measurement. In this study, we used a large cross-sectional sample of flight attendants to test for further empirical evidence distinguishing depression and suffering. Three key findings emerged. First, we found that both depression symptoms and suffering were correlated with worse well-being on all indices that were examined, but there was some variation in the magnitude of correlations found for each. Second, the results supported a large positive correlation between depression symptoms and suffering. However, there was evidence of notable non-concurrent depression and suffering, with more than 50% of participants reporting mild-severe suffering when depression symptoms were none-minimal and close to 30% endorsing mild-severe depression in the absence of suffering. Third, analyses involving categorical combinations of depression and suffering indicated that well-being was consistently lower among participants with concurrent moderate-severe depression and moderate-severe suffering. Similarly, when we focused on the subsample of participants who reported moderate-severe depression, well-being was generally lowest among participants with concurrent moderate-severe depression and severe suffering. Taken together, our findings suggest that depression and suffering are related and frequently co-occur, but they are distinct forms of distress that may be particularly disruptive to well-being when experienced concurrently.

This study extends existing research that has reported on associations of both depression symptoms and suffering with auxiliary variables by examining a range of indices across multiple dimensions of well-being (i.e., physical health, emotional well-being, psychological well-being, character strengths, social well-being, financial/material well-being). Consistent with prior studies (e.g., Büchi et al., 2009; Streffer et al., 2009), our findings suggest that depression symptoms and suffering differ in the strength of their associations with many indices of well-being. Compared to depression symptoms, suffering evidenced slightly stronger correlations with worse well-being on indices that offer a relatively pure assessment of physical health (e.g., general health, physically unhealthy days). Depression symptoms were more strongly correlated with worse well-being on almost all other indices, including some indices of physical health that also have a psychological component to them (e.g., vitality days, sleepless days). Although the FAHS dataset does not contain information about sources or objects of suffering, one potential explanation for this pattern of findings is that participants may have been inclined to respond to the broad global suffering item by reflecting primarily on their physical health. Additional research is needed to explore this possibility further, along with the more general question of whether the associations of suffering with different indices of well-being vary based on the object of suffering (e.g., physical pain, loneliness).

The findings of this study align with previous research that has shown evidence of non-concurrent depression and suffering (e.g., Wilson et al., 2007), highlighting the importance of assessing depression and suffering using measures that avoid conflating these two forms of distress. This will be an essential part of developing a robust body of empirical research on the causes of suffering, its unique implications for well-being, and interventions to support people who are suffering. Given that nearly 30% of participants with mild-severe depression were not suffering at all, further progress toward these ends may be achieved by ensuring that depression and suffering are not conflated with one another in language (e.g., “suffering from depression”)^1^ and measurement. Our findings also provide useful insight into the subjective experiences of suffering outside of clinical contexts, particularly among individuals who are not dealing with functionally impairing physical health issues. Almost 75% of participants reported mild-severe suffering, indicating that even people who are able to fulfill their responsibilities in a physically demanding employment role might still be suffering at some level. In addition, more than 25% of participants with symptoms of depression that were below the standard cutoff for probable depression on the PHQ-9 (i.e., none-mild depression) reported moderate-severe suffering. Hence, some individuals might be in a state of considerable distress which may go undetected by tools that are widely used in clinical settings to screen for distress (see Rana et al., 2019).

A key contribution of this study are the findings indicating that concurrent depression and suffering may be especially disruptive to various dimensions of well-being. We found that all indices of well-being were lowest among participants with moderate-severe depression and moderate-severe suffering. When joint associations of moderate-severe depression and moderate-severe suffering were decomposed, the bulk of the variance in most of the criterion variables was attributable to moderate-severe depression alone. This suggests that under circumstances of limited resources, a suitable first-line therapeutic approach would be to provide those who have more severe concurrent depression and suffering with a treatment that is known to be effective for depression. However, interventions that target both depression and suffering may be preferable for supporting the well-being of individuals with concurrent depression and suffering, especially if there is a chance that unresolved suffering might increase the risk of depression recurring among those who are in remission.

### Practical implications

Our findings suggest that healthcare professionals would do well to screen for suffering among the clients they work with (VanderWeele, 2019), particularly practitioners who are based in settings where mental health problems (especially depression) might present more frequently. Special care should be taken to ensure that clients who are at increased risk of depression, have subsyndromal symptoms of depression, or meet clinical criteria for depression undergo screening for suffering. Greater sensitivity to clients’ subjective experiences of suffering, its perceived impact, and responses to suffering may assist practitioners with making more informed decisions about appropriate diagnoses, treatments, and referrals to other relevant healthcare practitioners (Cowden et al., 2021b). Understanding the etiology of a client’s suffering and the nature of what they are experiencing could be especially important when suffering co-occurs with depression, as the efficacy of treatment approaches for depression may depend on the extent to which suffering is also addressed.

### Limitations and future research directions

To our knowledge, this is one of the first studies to explore associations of depression, suffering, and a wide range of well-being indices in a non-clinical population of employed adults. Collectively, the results provide further support for theory and a small number of previous studies that have found evidence indicating that depression and suffering are related but unique forms of distress. However, our findings should be considered alongside selected methodological limitations. First, the results are based on cross-sectional data and cannot be used to make inferences about causality. For example, it is possible that low levels of well-being might be an upstream cause of depression and/or suffering rather than a downstream consequence of depression, suffering, or both. Consistent with recent calls that have been made to improve the quality of empirical evidence on subjective suffering (VanderWeele, 2019; Cowden et al., 2021b), more rigorous research is needed to advance our understanding of suffering and its implications for well-being. Longitudinal studies that track changes in depression symptoms and suffering over time could offer valuable insight into the sources of suffering that precipitate depression, the circumstances under which depression leads to suffering, and the effects of concurrent depression and suffering on subsequent health and well-being.

Second, our sample consisted of currently employed flight attendants who were mostly female, White, and residents of a few Western, educated, industrialized, rich, and democratic (WEIRD) countries (i.e., Canada, United Kingdom, United States). Although the flight attendant occupation is predominantly comprised of females (Węziak-Białowolska et al., 2020), further study is required to determine the generalizability of our findings to the broader population of flight attendants in both WEIRD and less WEIRD contexts. Additional evidence is also needed to ascertain whether the results are transportable to other populations, including non-clinical populations that could enrich existing knowledge about “everyday suffering.”

Third, the FAHS survey was designed to capture a broad range of health and well-being topics over a more in-depth assessment of a narrow set of topics. This enabled us to evaluate a wide range of well-being dimensions, but a drawback of the FAHS dataset is that we were limited to single-item measures of many variables. Our results were also based entirely on self-report survey responses, which can be a potential source of bias. Future research might build on our findings by integrating objective markers of functioning (e.g., structured clinical interviews to diagnose depression) into measurement procedures and using multi-item measures to more comprehensively capture the conceptual breadth of criterion variables included in this study.

Fourth, similar to prior studies that have classified severity of suffering using a categorical approach (e.g., Wilson et al., 2007; Ruijs et al., 2014), we applied cutoffs to categorize participants into groups based on severity of suffering. Although our decision about cutoffs for severity of suffering was motivated by a combination of factors, including the interpretative advantage of maintaining some consistency with the number of categories and qualitative descriptors that are commonly applied to the PHQ-9, it is important to acknowledge that those cutoffs should not be viewed as prescriptive or definitive. Determining appropriate cutoff points for any measure of suffering will be a challenging process because it is a subjective experience that may not be reducible to a specific set of diagnostic criteria. However, future studies could explore the utility of alternative methods that may contribute to developing optimal reference standards for categories that correspond with varying degrees of suffering (see Rutjes et al., 2007).

## Conclusion

In summary, this study provided additional empirical evidence supporting a distinction between depression and the subjective experience of suffering. Although further research is needed to enrich our understanding of the conceptual similarities and differences between depression and suffering, our findings highlight the importance of screening for and attending to suffering as a form of distress that is distinct from depression. Practitioners are encouraged to develop competencies in assessing and effectively dealing with clients who are suffering, particularly those who report suffering concurrently with moderate-severe symptoms of depression.

## Data availability statement

The raw data supporting the conclusions of this article will be made available by the authors upon reasonable request.

## Ethics statement

This study was reviewed and approved by the Harvard Longwood Campus Institutional Review Board. The participants provided their written informed consent to participate in this study.

## Author contributions

RC was responsible for data analysis and writing the initial draft of the manuscript. TV provided analytic support. DW-B, EM, and TV assisted with reviewing and editing the manuscript. All authors contributed to the manuscript and approved the submitted version.

## Footnotes

1 A search in PsycINFO for peer-reviewed articles with the phrase “suffering from depression” in the title or abstract yielded 772 records published up to November 10, 2021, although there were few instances in which suffering itself was assessed.
