# Supplementary material for: Are depression and suffering distinct? An empirical analysis
Source: Front Psychol. 2022 Sep 2;13:970466. doi: 10.3389/fpsyg.2022.970466 (PMC9518749; doi:10.3389/fpsyg.2022.970466)
Supplement: Supplementary file 1 [file Data_Sheet_1.docx]

Are Depression and Suffering Distinct? An Empirical Analysis

**SUPPLEMENTAL FILE**

| Table S1  *List of Items Used to Assess Depression, Suffering, and Criterion Variables* | | |
| --- | --- | --- |
| Variable | Item/question | Response scale |
| **Depression**^a^ |  |  |
| PHQ-9_1 | Little interest or pleasure in doing things? | 0 (*Not at all*) to 3 (*Nearly every day*) |
| PHQ-9_2 | Feeling down, depressed, or hopeless? | 0 (*Not at all*) to 3 (*Nearly every day*) |
| PHQ-9_3 | Trouble falling or staying asleep, or sleeping too much? | 0 (*Not at all*) to 3 (*Nearly every day*) |
| PHQ-9_4 | Feeling tired or having little energy? | 0 (*Not at all*) to 3 (*Nearly every day*) |
| PHQ-9_5 | Poor appetite or overeating? | 0 (*Not at all*) to 3 (*Nearly every day*) |
| PHQ-9_6 | Feeling bad about yourself- that you are a failure and have let yourself or your family down? | 0 (*Not at all*) to 3 (*Nearly every day*) |
| PHQ-9_7 | Trouble concentrating on things, such as reading the newspaper or watching TV? | 0 (*Not at all*) to 3 (*Nearly every day*) |
| PHQ-9_8 | Moving or speaking so slowly that other people could have noticed? Or the opposite – being so fidgety or restless that you have been moving around a lot more than usual? | 0 (*Not at all*) to 3 (*Nearly every day*) |
| PHQ-9_9 | Thoughts that you would be better off dead or of hurting yourself in some way? | 0 (*Not at all*) to 3 (*Nearly every day*) |
| **Suffering** | To what extent are you suffering? | 0 (*Not suffering at all*) to 10 (*Suffering terribly*) |
| **Physical health** |  |  |
| General health^b^ | Would you say that in general your health is… | 1 (*Poor*) to 5 (*Excellent*) |
| Physically unhealthy days^b^ | Now thinking about your physical health, which includes physical illness and injury, for how many days during the past 30 days was your physical health not good? | 0 to 30 (*Days*) |
| Disability days^b^ | During the past 30 days, for about how many days did poor physical or mental health keep you from doing your usual activities, such as self-care, work, or recreation? | 0 to 30 (*Days*) |
| Vitality days^b^ | During the past 30 days, how many days did you feel very healthy and full of energy? | 0 to 30 (*Days*) |
| Sleepless days^b^ | During the past 30 days, how many days have you NOT gotten enough rest or sleep? | 0 to 30 (*Days*) |
| **Emotional well-being** |  |  |
| Mentally unhealthy days^b^ | Now thinking about your mental health, which includes stress, depression, and problems with emotions, for how many days during the past 30 days was your mental health not good? | 0 to 30 (*Days*) |
| Happiness^c^ | In general, how happy or unhappy do you usually feel? | 0 (*Extremely unhappy*) to 10 (*Extremely happy*) |
| Life satisfaction^c^ | Overall, how satisfied are you with life as a whole these days? | 0 (*Not at all satisfied*) to 10 (*Completely satisfied*) |
| **Psychological well-being** |  |  |
| Meaning in life^c^ | Overall, to what extent do you feel the things you do in your life are worthwhile? | 0 (*Not at all worthwhile*) to 10 (*Completely worthwhile*) |
| Sense of purpose^c^ | I understand my purpose in life. | 0 (*Strongly disagree*) 10 (*Strongly agree*) |
| **Character strengths** |  |  |
| Promote good^c^ | I always act to promote good in all circumstances, even in difficult and challenging situations. | 0 (*Strongly disagree*) 10 (*Strongly agree*) |
| Delay gratification^c^ | I am always able to give up some happiness now for greater happiness later. | 0 (*Strongly disagree*) 10 (*Strongly agree*) |
| **Social well-being** |  |  |
| Satisfying relationships^c^ | My relationships are as satisfying as I would want them to be. | 0 (*Strongly disagree*) 10 (*Strongly agree*) |
| **Financial and material well-being** |  |  |
| Financial stability^c^ | How often do you worry about being able to meet normal monthly living expenses? | 0 (*Worry all the time*) 10 (*Do not ever worry*) |
| Material stability^c^ | How often do you worry about safety, food, or housing? | 0 (*Worry all the time*) 10 (*Do not ever worry*) |
| *Note*. ^a^Patient Health Questionnaire-9 (Kroenke et al., 2001); ^b^Centers for Disease Control and Prevention (CDC) Health-Related Quality of Life 14 (HRQOL-14) (CDC, 2000); ^c^Secure Flourishing Index (VanderWeele, 2017). | | |

| Table S2  *Attributing Effects to Additive Interaction Between Moderate-severe Depression and Moderate-severe Suffering on Indices of Well-being (n = 4,652)* | | | |
| --- | --- | --- | --- |
| Criterion | Proportions of association attributable to main effects and interaction, % [95% CI] | | |
|  | Moderate-severe depression | Moderate-severe suffering | Moderate-severe depression × Moderate-severe suffering |
| **Physical health** |  |  |  |
| General health | 39.10% [35.06, 43.15] | 59.11% [55.81, 62.41] | 1.79% [-4.11, 7.69] |
| Physically unhealthy days | 16.00% [5.71, 26.29] | 85.21% [76.32, 94.10] | -1.21% [-15.00, 12.58] |
| Disability days | 21.42% [14.44, 28.40] | 52.48% [47.18, 57.78] | 26.10% [16.85, 35.36] |
| Vitality days | 73.98% [66.19, 81.78] | 50.66% [45.00, 56.31] | -24.64% [-35.35, -13.93] |
| Sleepless days | 74.23% [60.06, 88.41] | 35.78% [25.84, 45.72] | -10.02% [-28.46, 8.43] |
| **Emotional well-being** |  |  |  |
| Mentally unhealthy days | 39.65% [33.97, 45.32] | 25.33% [21.12, 29.55] | 35.02% [27.47, 42.57] |
| Happiness | 64.42% [59.67, 69.17] | 25.16% [21.78, 28.54] | 10.42% [4.01, 16.82] |
| Life satisfaction | 63.84% [58.87, 68.81] | 34.10% [30.50, 37.70] | 2.06% [-4.73, 8.85] |
| **Psychological well-being** |  |  |  |
| Meaning in life | 67.20% [61.11, 73.28] | 21.36% [17.06, 25.65] | 11.45% [3.26, 19.64] |
| Sense of purpose | 85.90% [77.98, 93.82] | 18.66% [13.30, 24.01] | -4.56% [-15.09, 5.97] |
| **Character strengths** |  |  |  |
| Promote good | 92.44% [81.51, 103.36] | 16.63% [8.86, 24.40] | -9.07% [-24.40, 6.26] |
| Delay gratification | 97.74% [85.10, 110.39] | 40.70% [31.42, 49.97] | -38.44% [-56.61, -20.27] |
| **Social well-being** |  |  |  |
| Satisfying relationships | 81.08% [73.22, 88.94] | 23.72% [18.31, 29.12] | -4.80% [-15.32, 5.73] |
| **Financial and material well-being** |  |  |  |
| Financial stability | 64.17% [54.93, 73.41] | 41.57% [34.75, 48.39] | -5.74% [-18.56, 7.08] |
| Material stability | 61.42% [52.74, 70.09] | 41.34% [34.91, 47.77] | -2.76% [-14.79, 9.27] |
| *Note*. CI = confidence interval. Proportions of associations attributable to individual and joint effects were determined using separate linear regression models in which each criterion variable was regressed on depression severity (none-mild depression = 0 vs. moderate-severe depression = 1), suffering severity (none-mild suffering = 0 vs. moderate-severe suffering = 1), and their interaction. All models adjusted for age, gender, sexual orientation, racial/ethnic status, marital status, child dependents, and adult/elderly dependents. Cumulative percentages within each row may not add up to 100% due to rounding. | | | |

| Table S3  *Estimated Marginal Means, Standard Errors, and Between-subjects Effect Sizes for Contrasts with the Moderate-severe Depression and No Suffering* *Group (n = 1,265)* | | | | |
| --- | --- | --- | --- | --- |
| Criterion | Moderate-severe depression & no suffering (*n* = 76) | Moderate-severe depression & mild suffering (*n* = 374) | Moderate-severe depression & moderate suffering (*n* = 500) | Moderate-severe depression & severe suffering (*n* = 315) |
| **Physical health** |  |  |  |  |
| General health |  |  |  |  |
| *EMM* ± *SE* | 3.66 ± 0.11 | 3.35 ± 0.06* | 2.91 ± 0.05*** | 2.50 ± 0.07*** |
| Cohen’s *d* [95% CI]^a^ | - | -0.16 [-0.27, -0.05] | -0.40 [-0.51, -0.29] | -0.59 [-0.71, -0.48] |
| Physically unhealthy days |  |  |  |  |
| *EMM* ± *SE* | 1.20 ± 1.01 | 3.73 ± 0.56* | 7.48 ± 0.51*** | 13.21 ± 0.60*** |
| Cohen’s *d* [95% CI]^a^ | - | 0.14 [0.03, 0.25] | 0.36 [0.25, 0.47] | 0.66 [0.55, 0.78] |
| Disability days |  |  |  |  |
| *EMM* ± *SE* | 0.68 ± 0.94 | 3.12 ± 0.53* | 7.66 ± 0.48*** | 14.24 ± 0.56*** |
| Cohen’s *d* [95% CI]^a^ | - | 0.15 [0.04, 0.26] | 0.43 [0.32, 0.54] | 0.80 [0.68, 0.91] |
| Vitality days |  |  |  |  |
| *EMM* ± *SE* | 8.90 ± 0.76 | 7.12 ± 0.43* | 5.44 ± 0.38*** | 4.46 ± 0.46*** |
| Cohen’s *d* [95% CI]^a^ | - | -0.13 [-0.24, -0.02] | -0.26 [-0.37, -0.15] | -0.32 [-0.43, -0.21] |
| Sleepless days |  |  |  |  |
| *EMM* ± *SE* | 14.10 ± 1.10 | 15.10 ± 0.62 | 16.71 ± 0.55* | 16.44 ± 0.66* |
| Cohen’s *d* [95% CI]^a^ | - | 0.05 [-0.06, 0.16] | 0.14 [0.03, 0.25] | 0.12 [0.01, 0.23] |
| **Emotional well-being** |  |  |  |  |
| Mentally unhealthy days |  |  |  |  |
| *EMM* ± *SE* | 5.71 ± 1.08 | 7.39 ± 0.61 | 12.21 ± 0.54*** | 17.51 ± 0.65*** |
| Cohen’s *d* [95% CI]^a^ | - | 0.09 [-0.02, 0.20] | 0.35 [0.24, 0.46] | 0.61 [0.49, 0.72] |
| Happiness |  |  |  |  |
| *EMM* ± *SE* | 6.22 ± 0.23 | 6.05 ± 0.13 | 5.32 ± 0.12*** | 5.03 ± 0.14*** |
| Cohen’s *d* [95% CI]^a^ | - | -0.04 [-0.15, 0.07] | -0.22 [-0.33, -0.11] | -0.28 [-0.39, -0.17] |
| Life satisfaction |  |  |  |  |
| *EMM* ± *SE* | 6.29 ± 0.25 | 6.20 ± 0.14 | 5.57 ± 0.13* | 4.86 ± 0.15*** |
| Cohen’s *d* [95% CI]^a^ | - | -0.02 [-0.13, 0.09] | -0.17 [-0.28, -0.05] | -0.32 [-0.43, -0.20] |
| **Psychological well-being** |  |  |  |  |
| Meaning in life |  |  |  |  |
| *EMM* ± *SE* | 7.40 ± 0.27 | 6.85 ± 0.15* | 6.22 ± 0.14*** | 6.26 ± 0.16*** |
| Cohen’s *d* [95% CI]^a^ | - | -0.11 [-0.22, -0.00] | -0.25 [-0.36, -0.14] | -0.23 [-0.34, -0.12] |
| Sense of purpose |  |  |  |  |
| *EMM* ± *SE* | 6.43 ± 0.32 | 5.99 ± 0.18 | 5.62 ± 0.16* | 5.98 ± 0.19 |
| Cohen’s *d* [95% CI]^a^ | - | -0.08 [-0.19, 0.03] | -0.14 [-0.25, -0.03] | -0.08 [-0.19, 0.03] |
| **Character strengths** |  |  |  |  |
| Promote good |  |  |  |  |
| *EMM* ± *SE* | 7.58 ± 0.23 | 7.55 ± 0.13 | 7.40 ± 0.12 | 7.65 ± 0.14 |
| Cohen’s *d* [95% CI]^a^ | - | -0.01 [-0.12, 0.10] | -0.04 [-0.15, 0.07] | 0.02 [-0.09, 0.13] |
| Delay gratification |  |  |  |  |
| *EMM* ± *SE* | 6.84 ± 0.26 | 6.88 ± 0.15 | 6.80 ± 0.13 | 6.94 ± 0.16 |
| Cohen’s *d* [95% CI]^a^ | - | 0.01 [-0.10, 0.12] | -0.01 [-0.12, 0.10] | 0.02 [-0.09, 0.13] |
| **Social well-being** |  |  |  |  |
| Satisfying relationships |  |  |  |  |
| *EMM* ± *SE* | 4.93 ± 0.34 | 5.03 ± 0.19 | 4.56 ± 0.17 | 4.65 ± 0.20 |
| Cohen’s *d* [95% CI]^a^ | - | 0.02 [-0.09, 0.13] | -0.06 [-0.17, 0.05] | -0.05 [-0.16, 0.06] |
| **Financial and material well-being** |  |  |  |  |
| Financial stability |  |  |  |  |
| *EMM* ± *SE* | 4.66 ± 0.39 | 4.33 ± 0.22 | 3.75 ± 0.20* | 3.29 ± 0.23*** |
| Cohen’s *d* [95% CI]^a^ | - | -0.05 [-0.16, 0.06] | -0.14 [-0.25, -0.02] | -0.20 [-0.31, -0.08] |
| Material stability |  |  |  |  |
| *EMM* ± *SE* | 6.08 ± 0.38 | 5.37 ± 0.22 | 4.74 ± 0.19*** | 4.20 ± 0.23*** |
| Cohen’s *d* [95% CI]^a^ | - | -0.10 [-0.21, 0.01] | -0.20 [-0.31, -0.09] | -0.27 [-0.38, -0.16] |
| *Note*. *EMM* = estimated marginal mean, *SE* = standard error. Models adjusted for age, gender, sexual orientation, racial/ethnic status, marital status, child dependents, and adult/elderly dependents. ^a^The moderate-severe depression and no suffering group served as the reference group in each model. *Comparison group differs significantly from the moderate-severe depression & no suffering group at *p* < .05 before but not after Bonferroni correction; ***Comparison group differs significantly from the moderate-severe depression & no suffering group at *p* < .05 both before and after Bonferroni correction (the *p*-value cutoff for Bonferroni correction was .05/15 = .0033 for each criterion variable). | | | | |

References

Centers for Disease Control and Prevention. (2000). *Measuring healthy days: Population assessment of health-related quality of life*. U.S. Department of Health and Human Services. https://stacks.cdc.gov/view/cdc/6406

Kroenke, K., Spitzer, R. L., & Williams, J. B. (2001). The PHQ-9: Validity of a brief depression severity measure. *Journal of General Internal Medicine*, *16*(9), 606–613. https://doi.org/10.1046/j.1525-1497.2001.016009606.x

VanderWeele, T. J. (2017). On the promotion of human flourishing. *Proceedings of the National Academy of Sciences*, *114*(31), 8148–8156. https://doi.org/10.1073/pnas.1702996114
